# The Financial Crisis

**Published:** 1921-06-18

**Authors:** 


### The Financial Crisis.

### Hospital Affected by Exchange.

THE Italian Hospital is very seriously affected by the rate of exchange of Italian money. Recently 2,000 lire sent to the hospital by a bank in Italy has realised only £27, whereas in normal times the donation would have represented about £80. The hospital does, of course, not restrict its ministrations to Italians, but succours all nationalities. It should be noted that the out-patients' departments have not been closed.

### St. Thomas's Hospital.

ST. THOMAS'S Hospital will celebrate its fiftieth year of service on its present site, Queen Victoria having opened the building on June 21, 1871. Considerations of expense preclude any extensive or elaborate celebrations of the jubilee of the institution. The Duke of Connaught has kindly consented to present prizes to the successful students of the hospital on that day, and the Nightingale Training School Garden-party will take place in the afternoon. It is hoped that, perhaps, some generous donor or donors may, in honour of the occasion, feel impelled to lighten or entirely remove the load of debt which is so seriously crippling the hospital and which has caused 100 beds to be closed down.

### Building Operations Stopped.

THE Board of Governors of the Royal Waterloo Hospital have decided to discontinue building operations on the hospital extension. In view of the Treasurer's statement as to the financial position, no other course, it is considered, was possible. The architect estimates that the expenditure of an additional £3,000 will enable the building operations to be carried to such a point as to conserve satisfactorily the work already done. The trustees of Smith's Charities recently sent the hospital £500—double their usual subscription.

### King's College Hospital.

UNDER a new scheme at King's College Hospital, any regular subscriber of not less than one guinea upon the admission of himself, his wife, or child under sixteen as a paying patient in a private ward will be entitled to ask that any subscription paid since January 1, 1920, may be credited against his account with the hospital.

